# Synthesis and Application of Tackifying Dispersant Poly (Vinyl Alcohol-Acrylic Acid-Triallyl Cyanate)

**DOI:** 10.3390/polym14030557

**Published:** 2022-01-29

**Authors:** Xiaoyan Chen, Weizhi Huang, Bobing He, Yafeng Zhang

**Affiliations:** 1 College of Chemistry, Sichuan University, Chengdu 610065, China; cxyyyazj@163.com (X.C.); euihsm5029739@163.com (W.H.); 2 College of Materials Science and Engineering, Xihua University, Chengdu 610065, China; zyffcb@163.com

**Keywords:** thickener, dispersant, graphene

## Abstract

At present, the thickener market is relatively advanced. Only by imparting thickeners with new properties can they meet the needs of the current market. In this work, a new modified tackifying dispersant poly (vinyl alcohol-acrylic acid-triallyl cyanate) (PVA-AA-t) was prepared via alcoholysis of a random copolymer composed of vinyl acetate (VAc), acrylic acid (AA), and triallyl cyanate (TAC) by a one-step high-temperature solution polymerization in methanol, which was a relatively simple method. The structure of the polymer was characterized by FTIR and TG. FTIR proved the successful synthesis of PVA-AA-t, while TG showed the thermal stability of PVA-AA-t at around 100 °C. The excellent thickening properties of the PVA-AA-t were observed using a nano particle size analyzer and a rotary viscometer. The nano particle size analyzer showed that the PVA-AA-t particles swelled in water to nearly nine times their initial size. The rotary viscometer showed that the viscosity of PVA-AA-t in water increased significantly, while PVA-AA-t was sensitive to electrolytes and pH, which changed the polymer molecular chain from stretched to curled, resulting in a decrease in viscosity. In addition, the dispersion properties of PVA-AA-t and a common thickener as graphene (Gr) dispersants were compared. The results indicate that PVA-AA-t has very good compatibility with Gr, and can effectively disperse Gr, because of the introduction of weak polar molecules (VAc) to the polymer molecules, changing their polarity, meaning that it is possible to use PVA-AA-t in the dispersion of Gr and other industrial applications (such as conductive textile materials, Gr batteries, etc.) derived from it.

## 1. Introduction

As we know, thickeners can be used to improve fluid viscosity and fluid rheology [1,2]. Adding a small amount of thickener to the required thickening system can significantly increase the apparent viscosity of the system. At present, thickeners are widely used in oil exploitation [3], agriculture [4], food processing [5], daily chemical products [6], textile printing and dyeing [7], the pharmaceutical industry, and other fields [8,9,10,11]. With the development of industry, the variety of thickeners is gradually increasing, among which cellulose [12] and polyacrylic acid thickeners [8] are the most widely used. At present, with the thickener market being well established, domestic and foreign researchers have begun to devote themselves to the research of various modified thickeners.

Valeria et al. [13] reported a study on the rheological properties of polymer chains hydrophobically modified with a small amount of N, N-dialkyl acrylamide (N, N-dihexylacrylamide (DHAM) and N, N-dioctylacrylamide (DOAM)). Chang-E Zhou et al. [14] prepared an associative thickener by compounding two polyacrylate-based copolymers—cationic starch and polyacrylic acid—mediated by polyethylene glycol and a polyacrylamide crosslinker, which were used for digital printing of nylon carpet with enhanced performance. Abdelrahman et al. [15] reported that the thickener of biodegradable poly (lactic acid-methacrylic acid)-crosslinked polymer hydrogel was formulated in situ via a one-pot reaction employing polycondensation of lactic acid and methacrylic acid followed by free radical polymerization with N, N-methylene diacrylamide crosslinker. Tao Guan et al. [16] reported a polymerizable acrylic-type hydrophobically modified ethoxylated urethane (HEUR), which was end-functionalized by the reactive methacrylate group and hydrophobic octadecyl tail to significantly improve the viscoelastic behavior of waterborne systems such as coatings and inks.

This modified thickener has a remarkable thickening effect; however, due to the variety of raw materials, high price, and complex synthesis steps, its commercial application value is greatly limited. Meanwhile, researchers are rarely involved in the application of modified thickeners [17]. For example, applications in wearable and implantable conductive textile materials have increased in recent years [18,19,20], one method of which is preparing the graphene (Gr) finished onto the surface of the fabric via multiple impregnation [20,21]. However, the surface of Gr lacks groups that form a good combination with textile materials, showing the poor adhesion between Gr and the matrix, along with weak physical fastness, seriously affecting the product quality [22]. Therefore, in order to improve Gr-Gr and Gr-fabric adhesion, as well as enhance the conductivity, thickener and dispersant should generally be added to graphene slurry [23], while the addition of a dispersant (such as alkyl diphenyl ether sulfonate, naphthalene sulfonic acid condensate, etc.) will affect the flexibility of the fabric. Therefore, in this paper, from the perspective of reducing the use of dispersant while improving the adhesion of Gr, a modified tackifying dispersant was synthesized, which can not only maintain the thickening performance, but also effectively disperse Gr.

## 2. Materials and Reagent

Vinyl acetate (VAc), acrylic acid (AA), azodiisobutyronitrile (AIBN), anhydrous methanol, and sodium hydroxide were purchased from Chengdu Kelong Chemical Co., Ltd. (Chengdu, China). Triallyl cyanate (TAC) was obtained from Shanghai McLean Biochemical Technology Co., Ltd. Graphene (Gr) (Shanghai, China) powder was purchased from Jiangsu Xianfeng Nano Material Technology Co., Ltd. (Jiangsu, China). Except for the VAc—which requires distillation in order to remove the polymerization inhibitor before use—and AIBN, which needs recrystallization, the other reagents were used as received.

### 2.1. Synthesis of PVAc-AA-t

Random copolymerization of poly (vinyl acetate-acrylic acid-triallyl cyanate) (PVAc-AA-t) was carried out as follows: Typically, calculated amounts of VAc, AA, and TAC were added sequentially to methanol (100% of monomer mass) in a 500 mL four-necked round-bottomed flask equipped with a mechanical stirrer, condenser, and nitrogen inlet. After bubbling nitrogen for 30 min to remove oxygen, the reaction was carried out at 65 °C for 3 h, with methanol solution of 5% AIBN (5‰ of monomer mass) slowly added during this process, followed by continuous stirring for 2 h. In this process, the viscosity of the system increased gradually, releasing a lot of heat, and finally forming transparent polymer gel particles (PVAc-AA-t).

The polymerization of polyacrylic acid (PAA) was carried out as follows: Typically, AA (100 mL), water as a solvent (250 mL), and the oxidant potassium persulfate (KPS) (5‰ of monomer molar mass) were added sequentially to a 500 mL four-necked round-bottomed flask equipped with a mechanical stirrer, condenser, and nitrogen inlet. After bubbling nitrogen for 30 min to remove oxygen, the reaction was carried out at −2 °C for 5 h, with the aqueous solution of 1% reducing agent (NaHSO_3_-FeCl_2_) (where n(KPS): n(NaHSO_3_): n(FeCl_2_) = 5:1:1) slowly added during this process. Throughout this process, the viscosity of the system increased gradually, releasing a lot of heat and, finally, forming PAA (the polymerization process of poly (acrylic acid-triallyl cyanate) (PAA-t) was the same as that of PAA, except for TAC).

### 2.2. Alcoholysis of PVAc-AA-t

**Figure 1:**
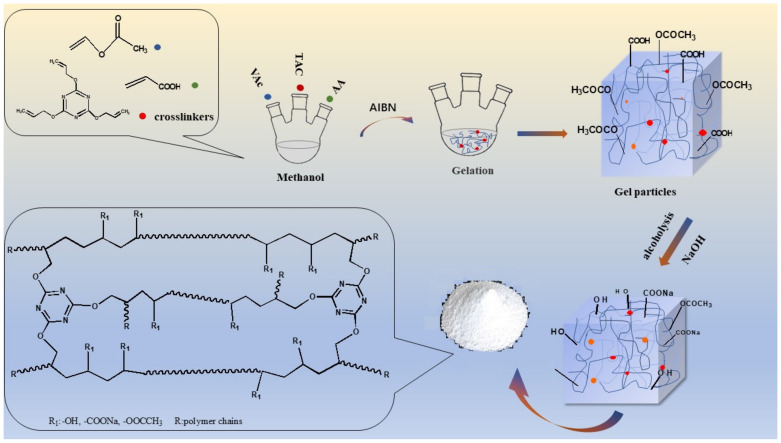
Flowchart of the reaction and alcoholysis of PVA-AA-t.

The gelatin particles synthesized in the reaction were added to a methanol solution of 0.5 mol/L NaOH at room temperature, with mechanical stirring throughout the whole process. Meanwhile, a methanol solution of 0.5 mol·L^−1^ NaOH was continuously added until the solution showed weak alkalinity, and could transform -OCOCH_3_ on the surfaces of gel particles into -OH, and transform -COOH into -COONa. In the alcoholysis stage, a lot of heat was produced, producing some colored substances because of side reactions if the temperature in the system was too high. Then, the obtained poly (vinyl alcohol-acrylic acid-triallyl cyanate) (PVA-AA-t) was rinsed with anhydrous methanol several times and dried in a vacuum at 50 °C until all of the solvents were removed. Finally, the dried samples were ground to an appropriate size by a ball mill, the reaction and alcoholysis of PVA-AA-t is shown in Figure 1. The alcoholysis of PAA and PAA-t was carried out in aqueous sodium hydroxide solution (0.5 mol·L^−1^). The specific operation steps were the same as for the alcoholysis of PVA-AA-t.

## 3. Characterizations

The viscosity of the PVA-AA-t aqueous solution (25 ± 1 °C) was measured using an NXS-11A viscometer at different shear rates; the formula is as follows:

(1) $$\mu =\frac{Τ}{\Gamma }\;$$

where *μ* is the viscosity (Pa·s), *Τ* is the shear stress (Pa), and *Γ* is the shear rate (s^−1^).

The FTIR spectra of the samples were recorded on a Nicolet iS50 Fourier-transform infrared spectrophotometer (Tokyo, Japan) using KBr pellets (frequency range from 4000 to 400 cm^−1^; each sample was scanned 32 times). In this experiment, the size and size distribution of the polymer were measured using a HELOS KR dry wet laser particle size analyzer produced by Sympatec GmbH (Clausthal-Zellerfeld, Germany). The polymer solid powder was tested by a dry method with compressed air. The sample pool was selected for wet testing of the polymer aqueous solution. The resistivity of the Gr electrode sheet was measured using an FT-340 series double-electrometric four-probe square resistance resistivity tester (ROOKO Instruments, Tokyo, Japan). Thermogravimetric analysis (TGA) was carried out on a TGA2 (METTLER TOLEDO, Columbus, OH, USA) to characterize the thermal stability of the samples. Samples (5–10 mg) were heated from room temperature to 500 °C at a heating rate of 10 °C·min^−1^.

## 4. Results and Discussion

**Figure 2:**
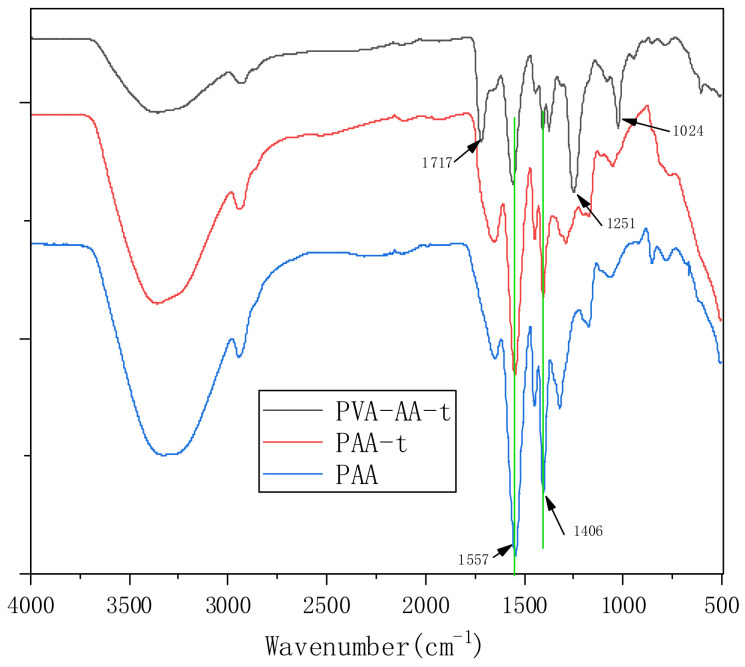
FTIR spectra of PVA-AA-t, PAA-t, and PAA (KBr).

FTIR measurement was employed to investigate the structure of PVA-AA-t, PAA-t, and PAA, as shown in Figure 2. In the three curves, the strong absorption bands at 3000–3500 cm^−1^ are assigned to -OH of H_2_O or VAc after alcoholysis (only in PVA-AA-t). The absorption bands at 2922 cm^−1^ should be assigned to C-H. The strong absorption bands 1557 cm^−1^ and 1406 cm^−1^ can be attributed to the symmetric and antisymmetric stretching vibration peaks of COO-, respectively. However, the weak absorption at 1717 cm^−1^ in the PVA-AA-t curve should also be noted, which represents the stretching vibration of carbonyl (-C=O) in the acetate group, indicating that the ester bond in the polymer molecule is incompletely alcoholized [24,25]; the C-O-C peaks of the ester bond at 1251 cm^−1^ and 1024 cm^−1^ also confirm this.

**Figure 3:**
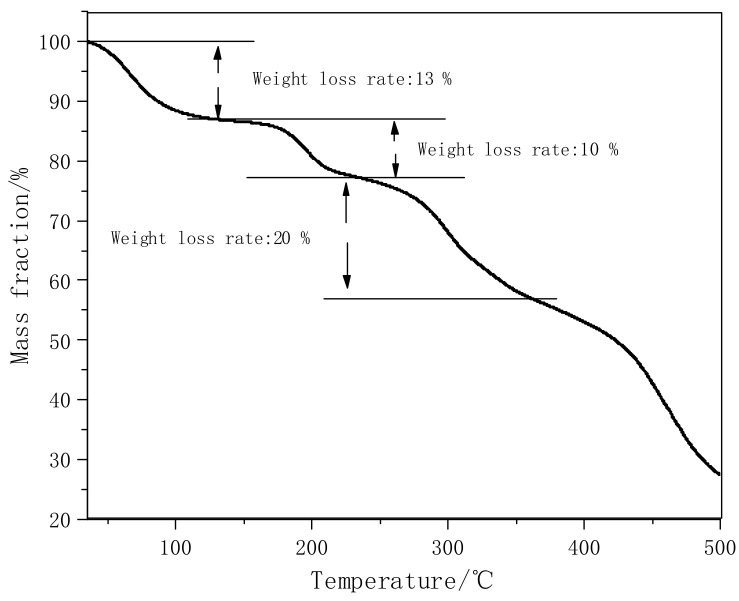
TG curve of PVA-AA-t under N_2_.

Figure 3 shows the TG curve of PVA-AA-t. Under a nitrogen atmosphere, the mass decreases for the first time at 50–100 °C, and the weight loss rate is 13%. This is mainly because the polymer absorbs moisture at room temperature. The weight loss rate of the polymer is 10% at 180–215 °C, because the carboxyl group in the polymer molecular chain is removed. The weight loss rate at 270–340 °C is 20%, which is caused by the breaking and decomposition of the ester or hydroxyl bonds in the molecular chain. When the temperature is higher than 430 °C, the mass of the polymer decreases rapidly, due to the fracture and decomposition of C-C in the main chain of the polymer. In general, the structure of the polymer is damaged when the temperature is higher than 180 °C, so it can be used in below 150 °C without damaging the properties of the polymer.

**Figure 4:**
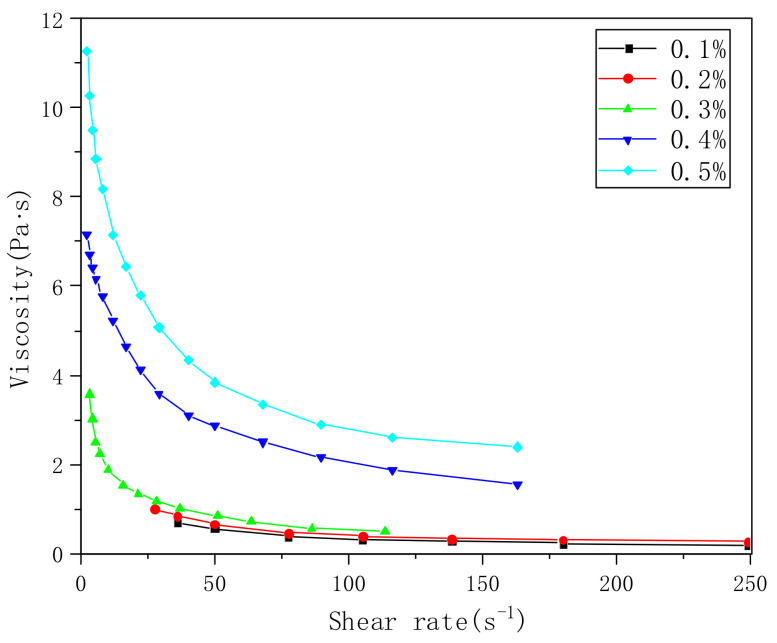
Viscosity curves of PVA-AA-t aqueous solutions with different concentrations.

We dissolved the PVA-AA-t in water and tested the viscosity, as shown in Figure 4. With the increase in the polymer’s concentration, the viscosity of the aqueous solution increases exponentially. When the concentration is less than 0.2%, the viscosity is less than 1 Pa·s (when the solution concentration is only 0.1%, there is almost no thickening effect). When the concentration increases to 0.3%, the viscosity increases rapidly to 4 Pa·s. The viscosity of 0.5% aqueous solution can reach 11 Pa·s, showing a clear thickening effect similar to that of other thickeners. Under shear force, with the increase in shear rate, the viscosity of the solution decreases rapidly, showing the characteristics of a pseudoplastic fluid.

**Figure 5:**
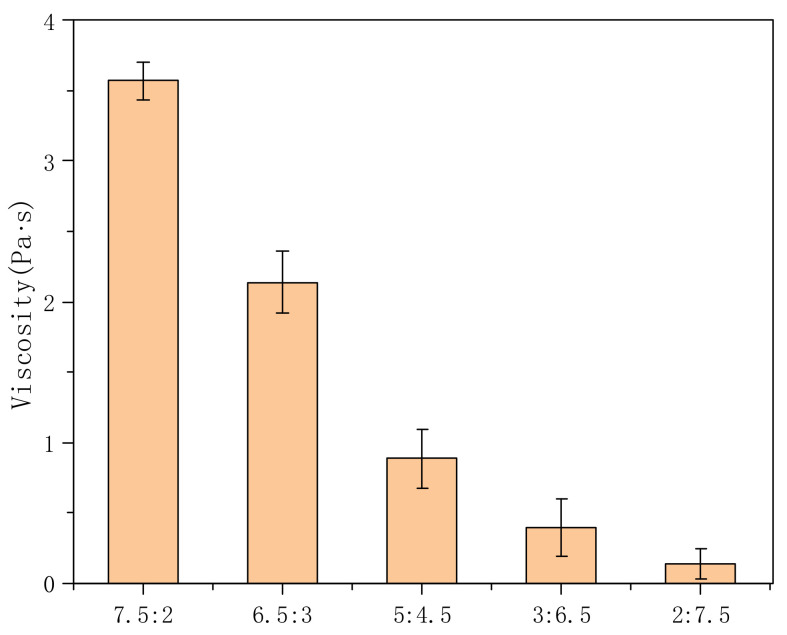
Effects of different monomer ratios on the viscosity of PVA-AA-t aqueous solution (concentration: 0.3%; TAC = 0.2 phr).

Figure 5 shows the variation in the viscosity of PVA-AA-t solutions with different monomer ratios. It can be seen from the figure that the viscosity is the highest when VAc:AA = 7.5:2, reaching 3.6 Pa·s. With the increase in the AA content of the copolymer, the initial viscosity decreases gradually. When the ratio of VAc:AA is 6.5:3, the initial viscosity drops to 2.14 Pa·s. When the ratio of VAc:AA is 2:7.5, the whole system has no thickening effect, showing Newtonian fluid characteristics. As can be seen from previous literature [16,26], sodium polyacrylate has been used as a thickener for many years; however, under these experimental conditions, we found that increasing the content of acrylic acid did not further improve the thickening effect—possibly due to the simultaneous copolymerization of three monomers in the system, affecting their respective reaction rates. Moreover, at high temperatures, the reaction rate of AA is faster than that of VAc, and the formed molecular chain is shorter, which leads to a poor thickening effect. On the other hand, VAc with a relatively slow reaction rate can form long-chain molecules at this temperature (65 °C).

**Figure 6:**
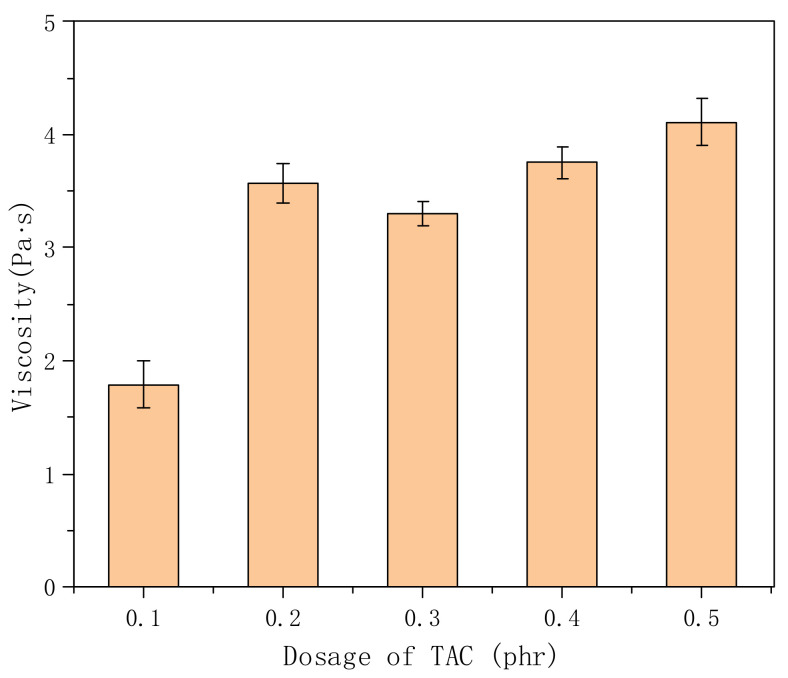
Effects of different amounts of crosslinking agent on the viscosity of polymer solution (concentration: 0.3%; VAc:AA = 7.5:2).

In this experiment, triallyl cyanate (TAC), which contains three ethylene groups, was used as a crosslinking agent. The crosslinking structure can be formed in the reaction process, and the more TAC is used, the higher the crosslinking density. From the Figure 6, we found that the viscosity (1.79 Pa·s) was relatively low when the dosage of TAC was 0.1 phr, which may have been due to the low dosage of the crosslinking agent, low crosslinking density of the molecular chain, and the polymer stretching and dissolving in water, resulting in the decrease in viscosity. Further increasing the amount of TAC, there was little change in viscosity, because TAC acts as a connecting point in the molecular structure [27], and the carboxyl and hydroxyl groups in the molecular chain play a thickening role. Therefore, from the perspective of application economy, the crosslinking agent is expensive; the dosage of TAC in the synthesis is 0.2 phr.

**Figure 7:**
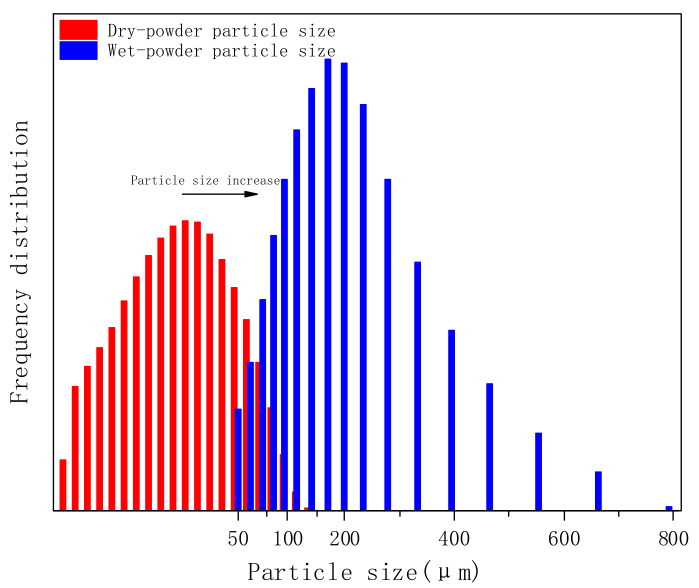
Particle size distribution of PVA-AA-t before and after swelling.

In this experiment, we compared the particle size distribution (PSD) of PVA-AA-t before and after swelling, as shown in Figure 7. The PSD of solid powder was in the range of 0.5–150 μm, the median diameter (D50) was 17.90 μm, and the average particle size was 24.74 μm (note: the solid polymer powder can be continuously ground by a ball mill, and the polymer powder within the fixed particle size range can be selected by standard sample separation). After swelling, the PSD of the polymer was in the range of 20–600 μm, D50 was 156.4 μm, and the average particle size was 187.2 μm. Comparing the two cases, the particle size increased nearly ninefold, showing an obvious tackifying effect. The swelling principle is the combination of non-associative thickening and associative thickening [28]; that is, -COO- in the polymer molecular chain combines with H_2_O to form hydrated ions, hindering the flow of molecules in the system, so as to achieve the purpose of thickening. On the other hand, the polymer molecular chain contains a small amount of acrylate chain, forming a comb-like structure. These hydrophobic short chains associate with one another to form a network structure, which can enhance the interaction between polymer particles and further increase the viscosity in water.

**Figure 8:**
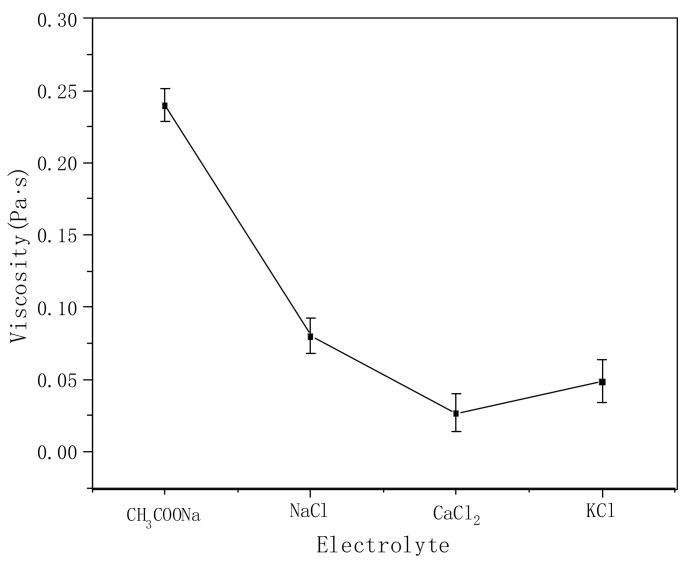
Effects of different electrolytes on the viscosity of polymer aqueous solution (polymer concentration: 0.3%, electrolyte concentration: 0.001 mol/L).

Figure 8 shows the effects of different electrolytes on the viscosity of the polymer solution. Under the same conditions, the viscosity decreases significantly (the original viscosity is nearly 4 Pa·s) after adding electrolytes. This is because the electrolyte can partially shield the carboxyl anion on the polymer molecular chain, resulting in the curling of the stretched macromolecular chain formed by the repulsion from the anions, reducing the friction between the molecular chains, and causing the viscosity to decrease rapidly with the addition of the electrolyte. The influence of different electrolytes on the viscosity follows the sequence CaCl_2_ > KCl > NaCl > CH_3_COONa. The greater the charge number of divalent ions at the same mole number, the stronger the shielding effect on the polymer molecules [29]. Compared with K^+^ and Na^+^, the larger the ion radius, the better the shielding effect on the charge of the molecular chain, making the molecular chain curl further and the viscosity decrease simultaneously. CH_3_COONa is a strong base and a weak acid salt. In aqueous solution, CH_3_COO- hydrolyzes to form CH_3_COOH, which weakens the shielding effect on the polymer molecular chain compared with the other three electrolytes. In general, the addition of an electrolyte has a great influence on the thickening effect of PVA-AA-t.

**Figure 9:**
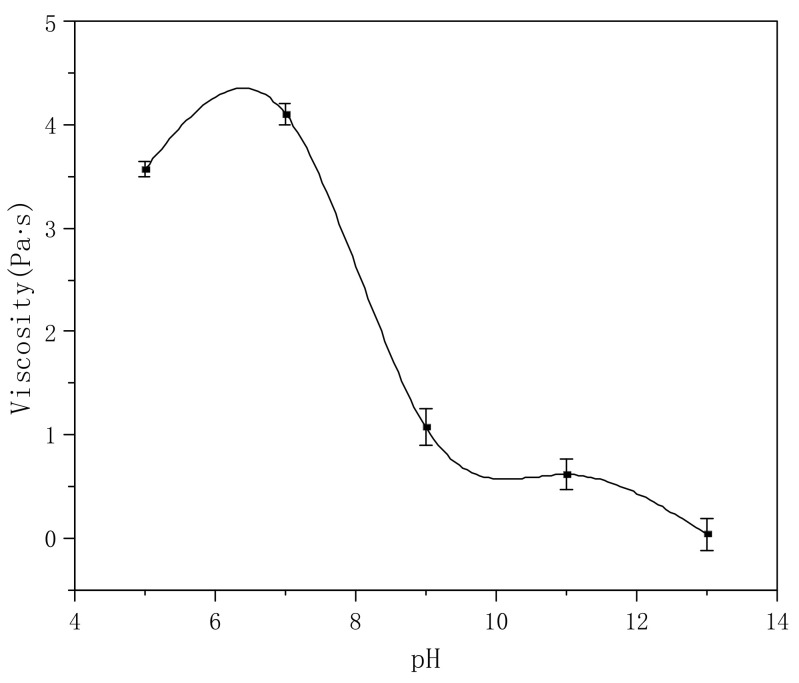
Effect of pH value on viscosity (polymer concentration: 0.3%).

There is a carboxylic sodium salt on the polymer molecules, the pH value of which has a great influence on the viscosity of the solution. Therefore, we compared the effects of different pH values on solution viscosity. As shown in Figure 9, the viscosity of the polymer solution (about 4.0 Pa·s) is the highest in the range of pH = 5–7; with the increase in the pH value, the viscosity begins to decrease gradually. When pH = 9, the viscosity decreases to 1.2 Pa·s; further increases in the pH value cause the viscosity to further decrease. When the pH is ~13, the whole solution has no thickening effect—this is mainly because under strong alkaline conditions, the system contains a large amount of ions, which weaken the hydration of the polymer molecular chain to H_2_O, making the molecular chain return to a curled state, reducing the intermolecular friction, and causing the decrease in viscosity.

**Figure 10:**
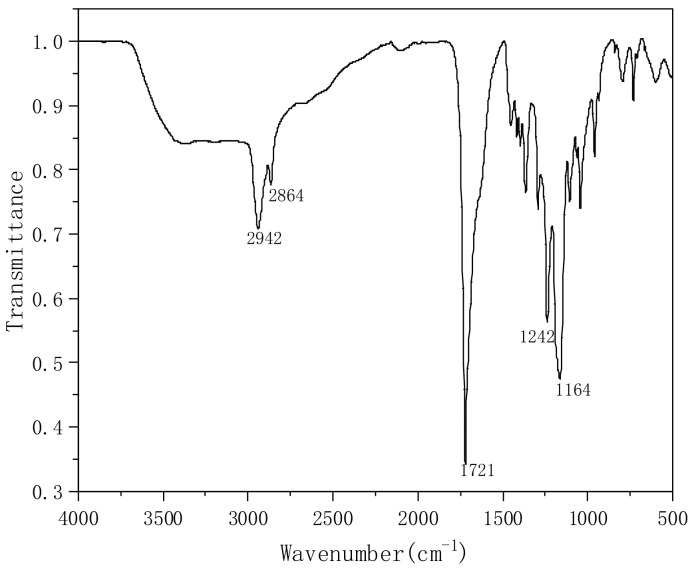
Infrared absorption of white turbidity after acidification.

On the other hand, when the pH value is reduced, white turbidity begins to appear in the solution. With the decrease in pH value, the sediment increases. We centrifuged the solution to obtain white insoluble matter, which was analyzed by FTIR, as shown in Figure 10. The peaks at 2942 cm^−1^ and 2864 cm^−1^ correspond to the symmetrical stretching vibration peaks of the C-H bond, while 1721 cm^−1^ is the carbonyl peak of the ester bond C=O, but the peak strength is significantly higher than the infrared absorption peak before acidification. It is possible that the carboxylic acid in the molecular chain reacts with the hydroxyl group to form ester bonds during acidification. The C-O-C peaks at 1242 cm^−1^ and 1164 cm^−1^ demonstrate the formation of ester bonds in the molecular chain. It is also possible that the peak intensity increases due to the increase in the carbonyl peak in the acidified carboxyl group, and the infrared absorption peaks of the acidified sample at 1557 cm^−1^ and 1408 cm^−1^ disappear, which may indicate that Na^+^ in the carboxylate is replaced by H^+^. The above research shows that PVA-AA-t has excellent thickening performance, and is sensitive to electrolytes and pH.

**Figure 11:**
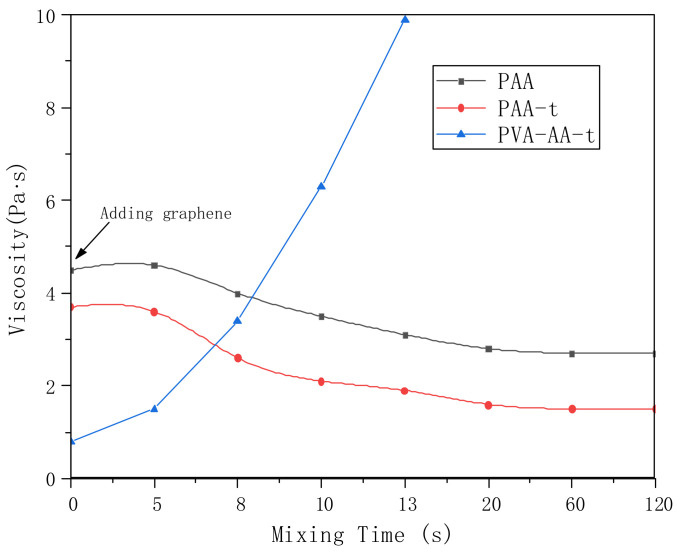
The changes in the viscosity of graphene dispersion slurry (polymer concentration: PAA: 1%; PAA-t: 1%; PVA-AA-t: 0.1%; Gr: 0.5%).

**Figure 12:**
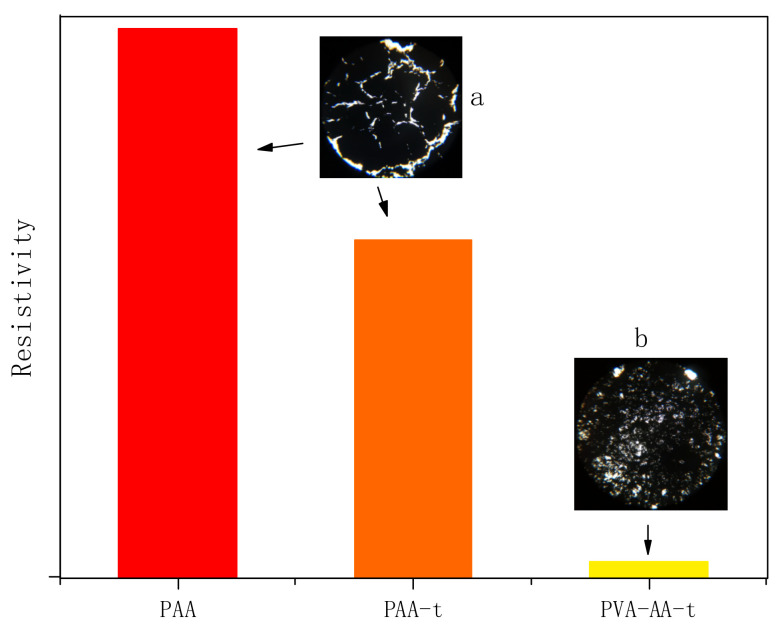
Resistivity of graphene electrode sheet. (**a**) PAA and PAA-t; (**b**) PVA-AA-t.

In addition, we studied the dispersion of Gr by PVA-AA-t. Gr was added to the polymer solution for high-speed stirring and dispersion, and the change in the system’s viscosity was measured using a portable viscometer (model: VL7-100B-d21-TS). At the same time, the dispersibility of Gr by PAA and PAA-t was compared. As shown in Figure 11, the initial viscosity of the three thickeners (PAA, PAA-t, and PVA-AA-t) was 4.3 Pa·s, 3.6 Pa·s, and 0.8 Pa·s, respectively. When t = 0 s, 0.5% Gr was added to three solutions. With the increase in dispersion time, the viscosity of the PAA solution decreased to a certain extent, and then remained stable. After long-time dispersion and grinding, there was still a large amount of agglomerated Gr in the PAA solution. This phenomenon also appeared in the PAA-t system, the viscosity of which decreased after the addition of Gr, with a large amount of agglomerated Gr. Conversely, when Gr was added to PVA-AA-t, the viscosity increased rapidly to more than 10 Pa·s under high-speed stirring, showing paste-like characteristics without fluidity. After full grinding and dispersion, the Gr slurry appeared as a dark glossy paste. Three kinds of Gr slurry were evenly coated on silicon wafers and glass (thickness: 60 μm) with an applicator, before being transferred to a vacuum-drying oven and dried at 60 °C for 5 h. The dried silicon wafers were used to measure the resistivity. The dried glass sheets were placed under a microscope (YKP-700C magnification: ×600) for observation (as shown in Figure 12a,b). As shown in Figure 12, the Gr dispersed by PVA-AA-t showed good resistivity, while the Gr dispersed by the other two thickeners (PAA, PAA-t) showed poor resistivity; this is because the Gr dispersed by PVA-AA-t was evenly distributed, without obvious agglomeration. After the Gr slurry was dried, it can be seen from Figure 12b that it showed evenly distributed pores (left by the dried polymer powder particles), and the Gr particles were overlapped with one another, showing good adhesion and good conductivity. However, the Gr dispersed by PAA and PAA-t had a lot of agglomeration, and the existence of the acrylic acid on the polymer chains likely inhibited bridging between particles, causing the force between the Gr particles to weaken, yielding obvious cracking and even falling off after drying, which affected the conductivity of the Gr electrode sheet.

The Gr in dispersed systems is stabilized mainly by charge stabilization and steric stabilization. The Gr surface contains a small amount of hydroxyl, carboxyl, and other functional groups, and is generally shown as an inorganic powder with weak polarity (except some with special chemical treatment). Therefore, a widely used sodium polyacrylate dispersant—such as PAA or PAA-t—in which the polymer chains contain acrylic acid only, has poor compatibility with Gr. If other components, such as alkyl acrylate, are introduced into a polymer such as PVA-AA-t, the second component will influence the adsorption of the polymer and the charge density of the particles adsorbed by it, consequently increasing the ability of that polymer to disperse Gr. In addition, the introduction of TAC makes the linear polymer molecules form a crosslinking structure, causing the polymer to form gel particles. When mixing and dispersing Gr, gel particles reduce the time needed for disentanglement between the long-chain polymers, and improve the dispersion efficiency Gr. After drying, the Gr powders are connected by dehydrated gel particles (PVA-AA-t), showing better adhesion and better electrical conductivity.

## 5. Conclusions and Perspectives

In this paper, a weakly polar polymer (PVA-AA-t) was synthesized by a relatively simple solution polymerization method. The polymer has similar properties to a alkali-soluble polyacrylic acid thickener, is sensitive to pH and electrolytes, and shows excellent thickening performance under neutral conditions, where the solution viscosity of only 0.3% PVA-AA-t can reach 4 Pa·s. In addition, it shows much better dispersion and thickening performance for Gr than other thickeners. Based on the findings of this paper, there is realistic potential for the application of Gr slurry dispersed by PVA-AA-t in the development of various products (such as conductive textile materials, robots, etc.) and industrial production for solving Gr adhesion, while the polymer could be used in research on the dispersion of other non-polar powders using PVA-AA-t.

## Data Availability

Data that support the findings of this study are included in the article.

## References

[1] Fu M., Huang Q., Gu Y., Xu L., Chen L. (2020). Development of Novel Silicon-Based Thickeners for a Supercritical CO_2_ Fracturing Fluid and Study on Its Rheological and Frictional Drag Behavior. Energy Fuels.

[2] Zhang Y., Zhu Z., Tang J. (2021). Investigation on modified polyether as an efficient CO_2_ thickener. New J. Chem..

[3] Zhao M., Li Y., Xu Z., Wang K., Gao M., Lv W., Dai C. (2020). Dynamic cross-linking mechanism of acid gel fracturing fluid. Colloids Surf. A Physicochem. Eng. Asp..

[4] Saade E., Fadhilah S.H., Kalsum U., Usman N.G. (2020). The effect of various processed seaweed, *Kappaphycus alvarezii* products as gel diet thickener on the utilization of nutrition in Rabbitfish, *Siganus guttatus* cultivation in the floating net cage. IOP Conference Series: Earth and Environmental Science.

[5] Rahman A., Genisa J., Zainal Z. (2020). Maltodextrin quality prepared from spoiled and leftover rice. IOP Conference Series: Earth and Environmental Science.

[6] Porfir’ev Y.V., Popov P.S., Zaichenko V.A., Shavalov S.A., Kotelev M.S., Kolybel’skii D.S., Tonkonogov B.P. (2019). Effect of Thickeners on Low-Temperature Greases. Chem. Technol. Fuels Oils.

[7] Chaudhary H., Singh V. (2018). Eco-Friendly Tamarind Kernel Thickener for Printing of Polyester Using Disperse Dyes. Fibers Polym..

[8] Li Z., Yuan X., Cui X., Liu X., Wang L., Zhang W., Lu Q., Zhu H. (2017). Optimal experimental conditions for Welan gum production by support vector regression and adaptive genetic algorithm. PLoS ONE.

[9] Hajighasem A., Kabiri K. (2015). Novel crosslinking method for preparation of acrylic thickener microgels through inverse emulsion polymerization. Iran. Polym. J..

[10] Hakim L., Triwitono P., Marseno D.W. (2021). Physicochemical characterization of Cocoyam (*Xanthosoma sagittifolium*) starch from Banjarnegara highland as a local source of carbohydrate. IOP Conference Series: Earth and Environmental Science.

[11] Saade E., Solicha A., Fadillah I.R. (2020). Effect of seaweed, *Kappphycus alvarezii* fermentation by various fermenters combinations as thickener on gel strength, attractiveness and palatability of gel diet in Tilapia, *Oreohromis niloticus*. IOP Conference Series: Earth and Environmental Science.

[12] Domínguez D., Blánquez A., Borrero-López A.M., Valencia C., Eugenio M.E., Arias M.E., Rodríguez J., Hernández M. (2021). Eco-Friendly Oleogels from Functionalized Kraft Lignin with Laccase SilA from *Streptomyces ipomoeae*: An Opportunity to Replace Commercial Lubricants. ACS Sustain. Chem. Eng..

[13] González-Coronel V.J., Jiménez-Regalado E. (2013). Effect of surfactant on the viscoelastic behavior of semidilute solution of two different families of water-soluble copolymers prepared by solution polymerization. J. Polym. Res..

[14] Zhou C.-E., Niu H., Zhang Q., Li H., Kan C.W., Sun C., Du J., Xu C. (2019). Preparation of an associative thickener for digital printing of nylon carpet. Pigment Resin Technol..

[15] Abdelrahman M.S., Nassar S.H., Mashaly H., Mahmoud S., Maamoun D., Khattab T.A. (2020). Polymerization products of lactic acid as synthetic thickening agents for textile printing. J. Mol. Struct..

[16] Guan T., Du Z., Peng J., Zhao D., Sun N., Ren B. (2020). Polymerizable Hydrophobically Modified Ethox-ylated Urethane Acrylate Polymer: Synthesis and Viscoelastic Behavior in Aqueous Systems. Macromolecules.

[17] Umar K., Yaqoob A.A., Ibrahim M.N.M., Parveen T., Safian M.T. (2021). Chapter Thirteen-Environmental Applications of Smart Polymer Composites. Smart Polymer Nanocomposites: Biomedical and Environmental Applications.

[18] Zheng S., Xu C., Zhang K., Yang X., Li R., Liu Y. (2020). Preparation and Application of Flexible Conductive Fabric Based on Silk. IOP Conference Series: Earth and Environmental Science.

[19] Zhang Y., Ren H., Chen H., Chen Q., Jin L., Peng W., Xin S., Bai Y. (2021). Cotton Fabrics Decorated with Conductive Graphene Nanosheet Inks for Flexible Wearable Heaters and Strain Sensors. ACS Appl. Nano Mater..

[20] Woltornist S.J., Alamer F.A., McDannald A., Jain M., Sotzing G.A., Adamson D.H. (2015). Preparation of conductive graphene/graphite infused fabrics using an interface trapping method. Carbon.

[21] Ren J., Wang C., Zhang X., Carey T., Chen K., Yin Y., Torrisi F. (2017). Environmentally-friendly conductive cotton fabric as flexible strain sensor based on hot press reduced graphene oxide. Carbon.

[22] Yaqoob A.A., Ibrahim M.N.M., Ahmad A., Reddy A.V.B. (2021). Toxicology and environmental application of carbon nanocom-posite. Environmental Remediation Through Carbon Based Nano Composites.

[23] Cataldi P., Ceseracciu L., Athanassiou A., Bayer I.S. (2017). Healable cotton–graphene nanocomposite conductor for wearable electronics. ACS Appl. Mater. Interfaces.

[24] Jiang L., Yang T., Peng L., Dan Y. (2015). Acrylamide modified poly(vinyl alcohol): Crystalline and enhanced water solubility. RSC Adv..

[25] Moritani T., Kajitani K. (1997). Functional modification of poly (vinyl alcohol) by copolymerization: Modification with carboxylic monomers. Polymer.

[26] Wang Y., Hudson N., Pethrick R., Schaschke C. (2014). Poly(acrylic acid)–poly(vinyl pyrrolidone)-thickened water/glycol de-icing fluids. Cold Reg. Sci. Technol..

[27] Chang S., Ma A.W.K., Lai H. (2019). New Insight into the Preparation of Starch-Based Spherical Microgels with Tunable Volume. Starch Stärke.

[28] Hashizaki K., Umeda R., Miura M., Taguchi H., Fujii M. (2020). Preparation and Rheological Properties of Cross-linked Liposomes Using Hydroxypropylmethylcellulose Bearing a Hydrophobic Anchor. Yakugaku Zasshi J. Pharm. Soc. Jpn..

[29] Jong L. (2020). Poly(acrylic acid) grafted soy carbohydrate as thickener for waterborne paints. Mater. Today Commun..

